# Quality of life in young patients with acute type a aortic dissection in China: comparison with Marfan syndrome and non-Marfan syndrome

**DOI:** 10.1186/s12872-024-03740-2

**Published:** 2024-02-29

**Authors:** Xin-fan Lin, Lin-feng Xie, Zhao-feng Zhang, Jian He, Yu-ling Xie, Xiao-fu Dai, Liang-wan Chen

**Affiliations:** 1 Department of Cardiovascular Surgery, Fujian Medical University Union Hospital, Xinquan Road 29, Fuzhou, Fujian 350001 P. R. China; 2 Fujian Provincial Center for Cardiovascular Medicine, Fuzhou, Fujian P. R. China

**Keywords:** Acute type a aortic dissection, Marfan syndrome, Quality of life, Propensity score matching (PSM)

## Abstract

**Background:**

There is a paucity of Chinese studies evaluating the quality of life (QoL) in young acute type A aortic dissection (AAAD) patients with Marfan syndrome.

**Methods:**

Young adult AAAD patients (younger than 45 years old) underwent surgical treatment at our institution from January 2017 to December 2020 were consecutive enrolled. The hospital survivors completed 1 year of follow up. Patients were divided into two groups according to the presence or absence of Marfan syndrome (MFS). A 1:1 propensity score matching (PSM) with a caliper 0.2 was conducted to balance potential bias in baseline. The follow-up data were analyzed primarily for change in quality of life and anxiety status.

**Results:**

After PSM, 32 comparable pairs were matched. The baseline data were comparable and postoperative complications were similar between groups. In terms of SF-36 scale, the role physical, bodily pain, role emotional and mental health subscales were no significantly improved in MFS patients over time. At 1 year after discharged, the subscale of mental health and bodily pain were significantly lower in the MFS group than in the non-MFS group. In terms of HADS assessments, the level of anxiety in MFS patients was significantly higher than in non-MFS patients at 1 year after discharged.

**Conclusions:**

The QoL in young AAAD patients with MFS is lower than those without MFS after surgery. This may be associated with the uncontrollable persistent chronic pain and the uncertainty and concerns for the disease’s progression.

## Introduction

Marfan syndrome (MFS) is a hereditary, autosomal dominant disorder due to mutations in the fibrillin-1 gene, that affects connective tissue in multiple organs, most notably the eyes, skeleton, and aorta [1]. For those with MFS, acute type A aortic dissection (AAAD) remains a common occurrence [2], this may be related to faulty connective tissue increases the risk that the aorta will dissect or rupture, causing serious cardiovascular problems or sometimes sudden death [3]. Previous studies have revealed that the majority of AAAD cases occurs in individuals aged over 60 years, with only about 10% of the total cases diagnosed in patients at their 40s [4]. However, a large proportion of AAAD patients who develop the condition in their 40s have a connection to Marfan syndrome [5].

While many studies have explored the disease characteristics and prognosis of type A aortic dissection, to date little has been done to explore the difference of survival after AAAD surgery with or without MFS. Surgical repair for the acute type A aortic dissection surgery is inevitable and difficult, and postoperative complications cannot be completely avoided. This results in a long-lasting course of treatment with a significant reduced quality of life for patients. Especially for the young patient with acute type A aortic dissection with a long-life expectancy. Therefore, it is of great significance to further research the influence factors of quality of life after AAAD surgery. The aim of the present investigation was to compare the quality of life in young AAAD patients with and without MFS after surgery in China, and to find out the factors affecting the medium-term prognosis.

## Materials and methods

### Patient selection and data collection

From January 2017 to December 2020, young adult patients with AAAD underwent surgical treatment at our institution were selected. Based on the history and clinical signs, the patients were grouped into MFS group and non-MFS group. A propensity score matching analysis (1:1) was conducted to balance the baseline differences between groups. The diagnosis of AAAD was confirmed by the aortic CTA or trans-thoracic echocardiography (TTE) for all patients preoperatively and completed the questionnaire through outpatient visits or Internet and smartphone during follow up. At least one year of follow-up was conducted recorded in individual file. The inclusion criteria were the following: (1) the age range was greater than 18 years and younger than 45 years; (2) all the patients received emergency modified triple-branched stent graft for extensive arch repair; (3) MFS was diagnosed based on the Ghent-2 criteria [6], while participants in the non-MFS group did not comply with the Ghent-2 criteria; (4) all patients provided consent for follow-up and signed a full informed consent. Participants with other co-morbid severe diseases, including cancer, moderate to severe chronic renal insufficiency, uncontrolled heart failure and the presence of severe cognitive, visual or hearing impairments were excluded.

### Surgical technique

The procedures were performed with patients under general anesthesia. Median sternotomy was performed, and cardiopulmonary bypass was established by 2 venous cannulas through the right atrium and 2 arterial return cannulas placed in the femoral and right axillary arteries. The ascending aorta was clamped at the base of the innominate artery and transected just above the sinotubular junction. During the cooling stage, a proximal aortic procedure was done according to the condition of aortic root.

When core temperature cooling to 22 °C, selective cerebral perfusion via the right axillary artery was established. After we cross-clamped the left common carotid artery and innominate artery, we transected the ascending aorta at the base of the innominate artery. Through the transverse incision of the ascending aorta, the main graft of the triple-branched stent graft was inserted into the true lumen of the arch and proximal descending aorta, and then each sidearm graft was positioned one by one into the aortic branch. Once the main graft and sidearm grafts were properly positioned, the restraining strings were withdrawn and then the main graft and sidearm grafts were deployed. Finally, the end of the triple-branch stent and the proximal artificial polyester blood vessel were anastomosed continuously to complete the operation [7, 8].

### Questionnaires

#### Assessment procedures

The current study was performed with the approval of the Ethic Committee of our hospital and performed in strict accordance with the Declaration of Helsinki. All patients were informed about the study in detail and signed consent forms. SF-36 was used to assess quality of life and HADS (HAD-A and HAD-S) was used to assess levels of anxiety and depression of patients. Patients completed the discharge questionnaire within the week after discharge and the follow-up questionnaire one year after discharge.

#### Medical outcomes study 36-item short-form health survey

Quality of life (QoL) was measured by the Medical Outcomes Study (MOS) 36-item short-form health survey (SF-36), which is applicable in a wide range of types and severities of health conditions and in a variety of clinical populations [9]. The SF-36 consists of 36 questions, grouped into 8 multiple-item domains, including physical functioning (PF), role physical (RP), bodily pain (BP), general health (GH), vitality (VT), social functioning (SF), role emotional (RE) and mental health (MH) [10]. 2 standardized summary scores, the physical component summary (PCS) score and the mental component summary (MCS) score, were calculated for each subject using the SF-36 [11]. For each measure, a higher score suggests a higher QoL.

#### Hospital anxiety and depression scale

As a commonly used anxiety and depression assessing self-rating scale, the hospital anxiety and depression scale (HADS) is used to evaluate the psychological distress of nonpsychiatric patients [12]. It comprises 14 items, 7 of which measure anxiety (HADS-A) and another 7 measures depression (HADS-D). Each question has four alternative responses, with scores ranging from 0 to 3. The score is positively correlated with the severity of depression or anxiety [13].

### Statistical analysis

The statistical analysis was conducted in R software version 4.3.2. Perioperative and 1-year post discharge SF-36 scores and HADS scores of two groups were tested for normality, and the results showed normal distributions. Thus, it was expressed as the mean ± standard deviation and were compared among groups with an independent-sample t test. The categorical data were presented as percentage and were compared by chi-squared test. Propensity score matching (PSM) was calculated based on a logistic regression model, with a caliper 0.2, matching ratio = 1:1 to balance the baseline variables. The potential confounding variables being matched consisted of patients characteristic (age, male, BMI, marital status, education level, employment, residence) and admission status (SBP, heart rate, ascending aorta diameter, left ventricular ejection fraction, preoperative malperfusion, extent of AD involvement, concomitant CABG, root procedure). A *p* values < 0.05 were defined as statistical significance.

## Results

**Fig. 1:**
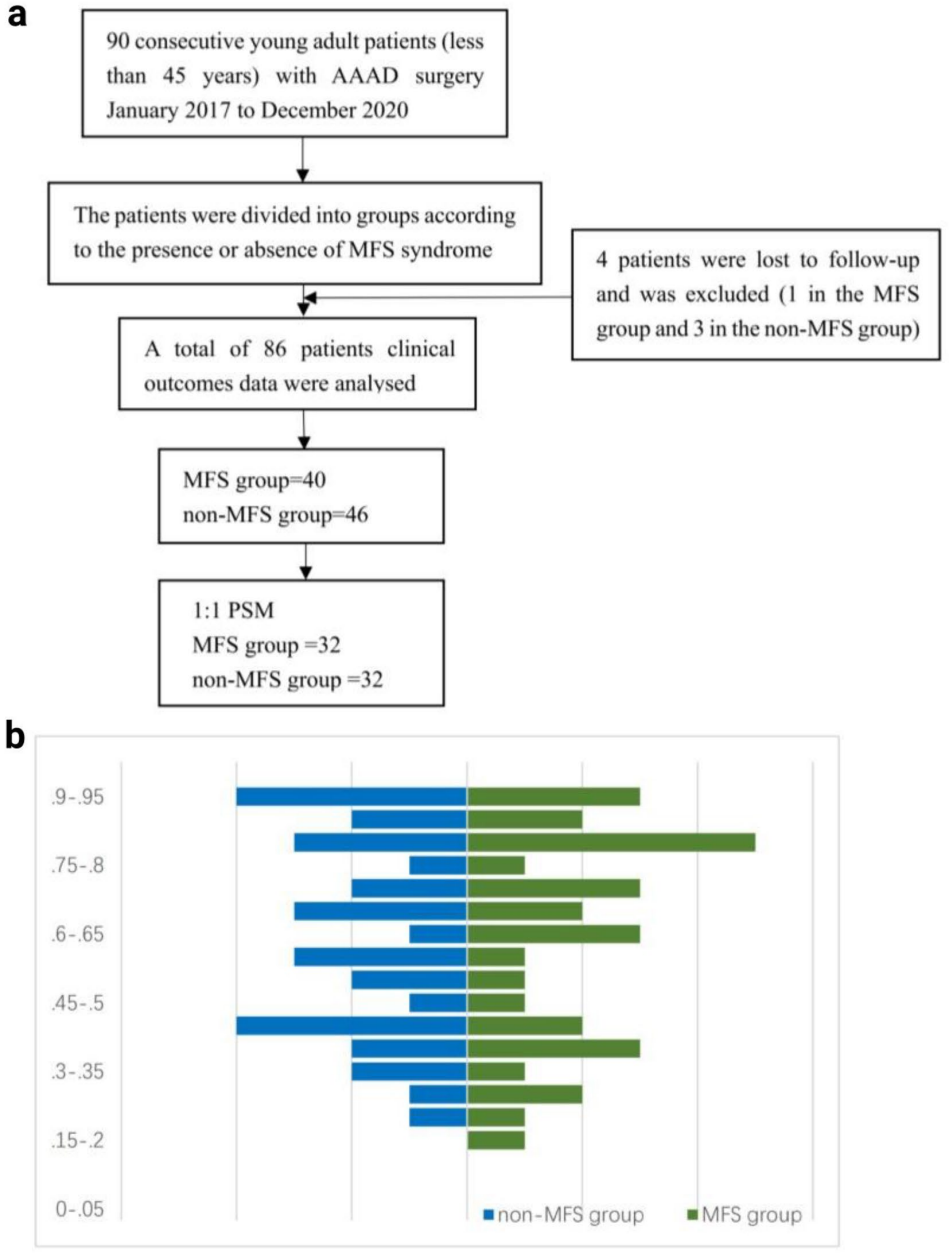
**a** Patient selection flowchart. **b** Mirror histogram of propensity scores for patients

The patient selection chart and PSM details are shown in Fig. 1. A total of 90 patients were recruited into the trial, with 86 completing the whole questionnaire (40 people in MFS group and 46 people in non-MFS group). 4 participants (1 in the MFS group and 3 in the non-MFS group) were lost to follow-up. To address the potential bias in baseline, a 1:1 propensity score matching (PSM) analysis was carried out. In the end, 32 comparable pairs were matched.

The baseline characteristic of the two groups before and after PSM are recorded in Table 1. Before matching, MFS patients were considerably younger (37.2 ± 13.5 years VS 43.6 ± 15.7 years, *p* = .047), with a lower mean BMI (21.8 ± 3.8 VS 26.7 ± 4.5, *p* < .001) and with a wider ascending aorta diameter (62.85 ± 8.15 mm VS 38.55 ± 9.58 mm, *p* < .001). While the non-MFS patients presented with a higher admission systolic blood pressure (135.5 ± 25.7 mmHg VS 119.3 ± 15.2 mmHg, *p* < .001). After PSM, there are still significant difference in ascending aorta diameter between two groups (53.8 ± 6.1 mm VS 45.5 ± 8.2 mm, *p* < .001). Apart from this, no other statistically differences were observed. No obvious differences were detected in postoperative outcomes in the patients between groups after matching. (Table 2)

**Table 1:** Baseline characteristic before and after propensity score matching

Parameters	Before PSM			After PSM		
	MFS group	non-MFS group	p	MFS group	non-MFS group	p
**Number**	40	46		32	32	
Age (years)	37.2 ± 13.5	43.6 ± 15.7	0.047	38.5 ± 11.7	41.7 ± 13.1	0.307
Male (n, %)	28 (70.0%)	31 (67.4%)	0.795	25	28	0.320
BMI (kg/m^2^)	21.8 ± 3.8	26.7 ± 4.5	< 0.001	22.9 ± 3.5	24.7 ± 3.8	0.053
Unmarried (n, %)	11 (27.5%)	12 (26.1%)	0.883	10	11	0.790
Education level (n, %)			0.529			0.435
Junior high school education or below	10 (15%)	15 (32.6%)		8	11	
High school diploma	28 (70%)	27 (58.7%)		22	18	
Undergraduate degree or higher	2 (5.0%)	4 (8.7%)		2	3	
Employment (n, %)			0.768			0.641
Unemployment	29 (72.5%)	30 (65.2%)		24	20	
Part time	9 (22.5%)	13 (28.3%)		7	10	
Full time	2 (5.0%)	3 (6.5%)		1	2	
Residence (n, %)			0.255			0.511
City	26 (65.0%)	36 (78.3%)		22	25	
Town	13 (32.5%)	8 (17.4%)		9	7	
Rural	1 (2.5%)	2 (4.3%)		1	0	
SBP (mmHg)	119.3 ± 15.2	135.5 ± 25.7	< 0.001	124.4 ± 13.9	130.3 ± 20.5	0.167
Heart rate (beats/min)	81.8 ± 10.5	76.8 ± 14.7	0.077	80.1 ± 10.1	78.5 ± 14.5	0.617
Ascending aorta diameter (mm)	62.9 ± 8.2	38.6 ± 9.6	< 0.001	53.8 ± 6.1	45.5 ± 8.2	< 0.001
Left ventricular ejection fraction (%)	64.4 ± 8.3	63.8 ± 7.9	0.864	62.5 ± 9.2	63.4 ± 7.2	0.664
Preoperative malperfusion (n, %)	5 (12.5%)	3 (6.5%)	0.341	4	2	0.391
Neurological deficit (n, %)	2 (5.0%)	2 (4.3%)	0.886	2	2	1.00
Extent of AD involvement (n, %)						
Root	15 (37.5%)	7 (15.2%)	0.085	12	6	0.273
Supra-aortic vessels	9 (22.5%)	5 (10.9%)	0.145	5	3	0.450
Abdominal aorta	30 (75.0%)	32(69.6%)	0.393	25	24	0.768
CABG (n, %)	2 (5.0%)	3 (6.5%)	0.764	2	3	0.641
Root procedure (n, %)			0.006			0.068
Valve sparing	10 (25.0%)	22 (47.8%)		8	15	
Bentall procedure	30 (75.0%)	24 (52.2%)		24	17	

Continuous variables were present as mean ± standard deviation (SD). Categorical variables were shown as number (%)

*AD* aortic dissection, *BMI* body mass index, *SBP* systolic blood pressure, *CABG* coronary artery bypass grafting

**Table 2:** Perioperative outcomes before and after propensity score matching

Item	Before PSM			After PSM		
	MFS group	non-MFS group	p	MFS group	non-MFS group	p
**Number**	40	46		32	32	
Neurologic dysfunction	3 (7.5%)	5 (10.9%)	0.869	3	2	0.641
Acute kidney injury	4 (10.0%)	8 (17.4%)	0.500	3	6	0.281
Hepatic dysfunction	2 (5%)	6 (13.0%)	0.363	2	5	0.230
Sepsis	0	1 (2.2%)	< 0.001	0	1	0.313
Tracheotomy	1 (2.5%)	1 (2.2%)	0.537	1	1	1.00
Low cardiac output syndrome	1 (2.5%)	1 (2.2%)	0.537	1	1	1.00
Sternal complications	2 (5%)	1 (2.2%)	0.901	2	1	0.554
Hospital time (days)	21.2 ± 9.9	19.9 ± 8.6	0.568	22.7 ± 8.2	20.1 ± 8.5	0.218
Hospital costs (1000 RMB)	165.7 ± 31.7	171.2 ± 34.5	0.223	163.4 ± 30.1	168.1 ± 32.9	0.553

Continuous variables were present as mean ± standard deviation (SD). Categorical variables were shown as number (%)

Table 3 analyzed the SF-36 scores and HADS scores at discharge and 1 year post discharge between groups after PSM. The QoL of the non-MFS patients were significant improved in all dimensions in SF-36 after 1-year discharged. However, in MFS patients, no obvious improvement was observed in RP, BP dimension of PCS and RE, MH dimensions of MCS over 1 year. In the comparison of HADS scores within MFS group after matching, the HADS-A score was 16.4 ± 4.0 at discharge and 12.0 ± 5.6 at 1 year post discharge (*p* = .001). The HADS-D score was 14.4 ± 6.0 and 12.6 ± 4.7, respectively (*p* = .186). In non-MFS group, the HADS-A score was 14.7 ± 5.0 at discharge and 7.7 ± 5.3 at 1 year post discharge (*p* < .001). The HADS-D score was 12.8 ± 5.1 and 7.2 ± 4.5, respectively (*p* < .001).

**Table 3:** SF-36 mean scores and HADS mean scores at discharge and at 1 year post discharge after propensity score matching

	SF-36								HADS	
	PF	RP	BP	GH	VT	SF	RE	MH	HADS-A	HADS-D
**MFS group**										
Discharge	62.4 ± 13.5	65.4 ± 15.1	61.7 ± 17.1	63.5 ± 13.7	60.1 ± 13.4	64.3 ± 15.4	64.1 ± 18.8	60.5 ± 11.1	16.4 ± 4.0	14.4 ± 6.0
1 year post discharge	71.9 ± 14.6	73.4 ± 19.5	66.2 ± 16.8	73.2 ± 16.5	69.3 ± 15.1	73.2 ± 17.5	69.7 ± 16.5	66.1 ± 14.2	12.0 ± 5.6	12.6 ± 4.7
***p***	0.009	0.071	0.292	0.014	0.012	0.035	0.210	0.084	0.001	0.186
**non-MFS group**										
Discharge	62.3 ± 14.7	65.3 ± 18.1	62.5 ± 15.7	66.1 ± 15.3	64.3 ± 14.8	64.9 ± 15.1	66.1 ± 15.5	65.7 ± 16.7	14.7 ± 5.0	12.8 ± 5.1
1 year post discharge	71.2 ± 15.6	74.3 ± 17.5	73.6 ± 14.9	75.5 ± 15.1	73.4 ± 16.4	72.9 ± 16.3	74.0 ± 14.3	74.6 ± 14.5	7.7 ± 5.3	7.2 ± 4.5
***p***	0.022	0.047	0.005	0.016	0.023	0.046	0.038	0.026	< 0.001	< 0.001

Continuous variables were present as mean ± standard deviation (SD)

*PF* physical functioning, *RP* role physical, *BP* bodily pain, *GH* general health, *VT* vitality, *SF* social functioning, *RE* role emotional, *MH* mental health, *HADS-A* hospital anxiety and depression scale-anxiety, *HADS-D* hospital anxiety and depression scale-depression

**Fig. 2:**
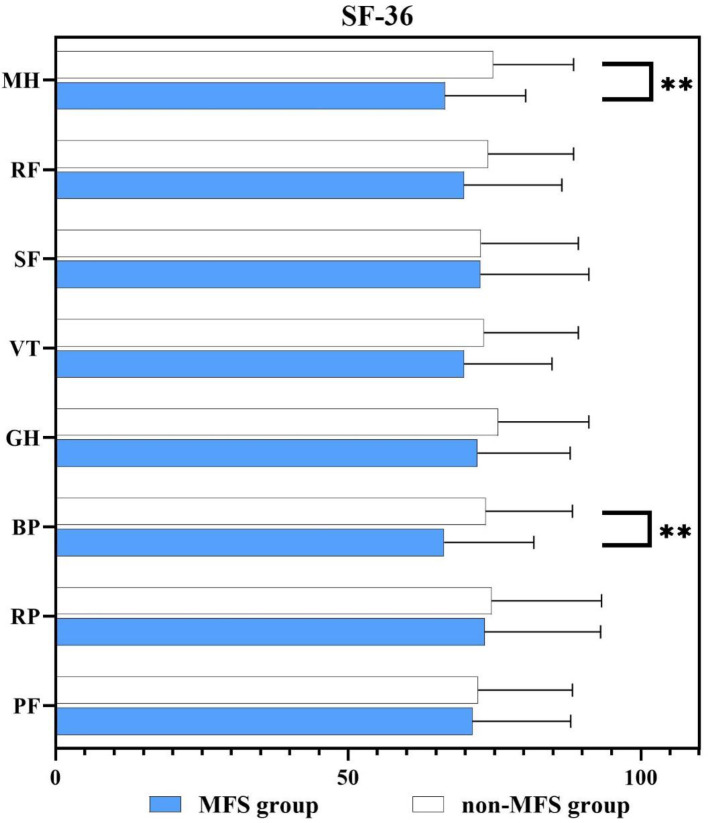
Comparison of the all dimensions of SF-36 scores at 1 year after discharge after PSM. **: *p* < .01. *PF* physical functioning, *RP* role physical, *BP* bodily pain, *GH* general health, *VT* vitality, *SF* social functioning, *RE* role emotional, *MH* mental health

**Fig. 3:**
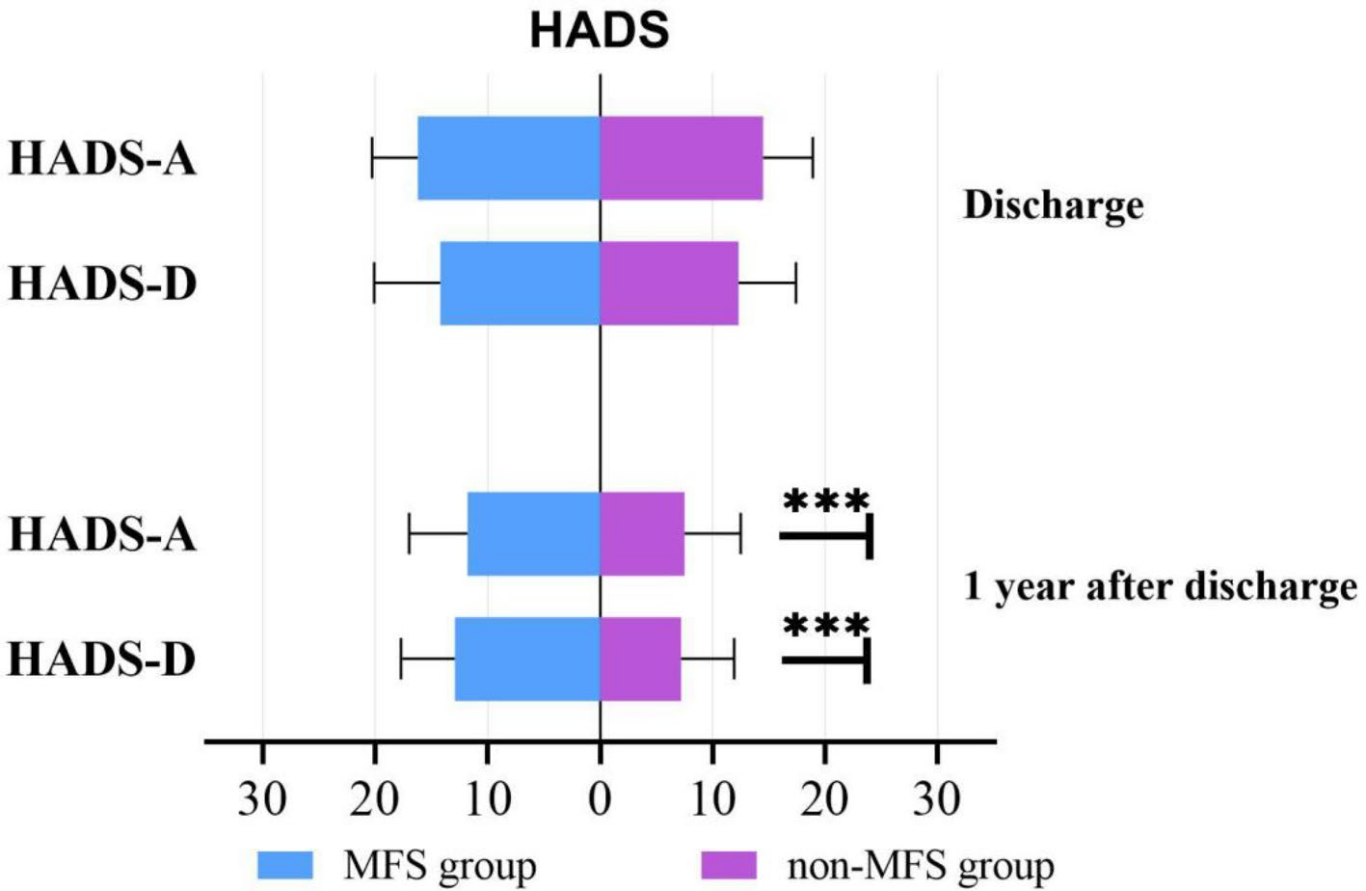
Comparison of HADS scores at different time points after PSM. ***: *p* < .001. *HADS-A* hospital anxiety and depression scale-anxiety, *HADS-D* hospital anxiety and depression scale-depression

Figure 2 Comparing the scores on each dimension of the SF-36 scores between groups at 1 year after discharge after PSM. The scores of all the eight subscales in MFS group were lower than non-MFS group, and there was a statistically significant difference (*p* < .01) in BP dimension of PCS and MH dimensions of MCS. Figure 3 While no significant difference was observed in HADS (both HADS-A and HADS-D) scores between groups at discharge, the MFS group HADS (both HADS-A and HADS-D) scores were much higher than non-MFS group at 1 year post discharge (*p* < .001).

## Discussion

The therapeutic strategies for aortic dissection will become even more diverse as novel surgical technique are becoming available. For the AAAD, should be kept as simple as possible, as the main goal is to save the life. Specifically for patients with MFS, the progressive aortic root dilatation and persistence of residual false lumen makes the likelihood of reoperation remains [14], might be the important factor affecting the long-term outcome. Considering the conditions above, we try to perform ascending aorta replacement combined with open triple branched stent graft placement in Marfan patients, the stent expanded and stabled the true lumen and obtained satisfactory early clinical results [15]. For the young patients with longer life expectancies. We also pay increasing attention to the postoperative QoL of patients in addition to clinical efficacy. It can comprehensively assess the overall health status of patients, to achieve the dual rehabilitation of physical and psychological factors [16]. For this aim, we designed this study, using the SF-36 and the HADS questionnaire to explore the quality of life in young AAAD patients with and without MFS after surgery in China.

Our study showed that patients with Marfan syndrome did not show significant improvement in the role physical, bodily pain, role-emotional, and mental health dimensions of quality of life at 1-year post-discharge. The phenomenon has not only been because of postoperative physical functioning deterioration, but also due to the psychological problems caused by the disease. In daily life, individuals with Marfan syndrome may experience new challenges in their family relationships, economic situation, and social interactions. Meanwhile, due to physical limitations, patients may also encounter obstacles in employment and access to insurance [17]. Compared to patients without MFS, those with MFS consistently scored lower in bodily pain assessments. Except for the postoperative pain, previous research has demonstrated that the percentage of MFS patients suffering from chronic pain ranges from 47 to 92% [18]. Meanwhile, the chronic pain is linked to profound disability and significant psychological burden. This is also reflected in the consistently elevated in the HADS score in our study. AAAD patients often have a certain extent depression, with high levels of anxiety during perioperative period. Improvements were obtained in non-MFS patients over time up to 1 year after the end of treatment. However, this situation is hardly occurring in MFS group, our results revealed that the improvement in anxiety levels was more pronounced compared to that in depression levels. The reason may be the continued emotional distress such as negative illness perceptions and decreased self-esteem, as well as negative cardiovascular consequences, including increased reoperation rate and premature mortality can lead to far-reaching implications for adverse psychological outcomes. Peters et al. found that over 40% of their study cohort had a significant degree of depressive symptoms [19]. It could have the strongest and most direct effects on QoL among all disease-related factors and was also long ignored as public health problem in the developing country.

Based on our results, we argue that the current treatment process targeting MFS patients this special population, has the following problems are given as enumerated below. Firstly, though, chronic pain is a significant and persistent problem in MFS, there is a lack of unified consensus on the proper postoperative chronic pain to use. At the same time, Nelsen et al. noted that very few patients received medical procedures for pain and less than a half of them were satisfied with their current pain treatment [20]. We will focus future efforts on the better management of postoperative MFS patients, could potentially improve an individual’s satisfaction with life by facilitating work participation and everyday activities [21]. Secondly, postoperative psychiatric problems of MFS patient have been neglected for long time. Patients with high level of anxiety always have poorer awareness of seeking medical care. To face such uncomfortable experiences, they just choose to self-medicate and drinking. While workarounds may be successful in symptoms- relieving in the short term, they can lead to longer-term complications, such as drug and alcohol abuse. A lack of early detection and earlier intervention is linked to an increased risk of depression. Postoperative mental health education and mental health service online after discharge can be the keyway to improve the quality of life of many young adult patients. psychological and social assessments and psychological guidance should provide at each follow-up.

In our survey, we found that young AAAD patients with MFS syndrome have the worse quality of life after surgery compared to the patients without MFS. This discrepancy is largely because of uncontrollable persistent chronic pain and uncertainty concerns for the disease’s progression. This suggests that treatment process and psychological interventions targeting the general patients have not worked as effectively for those with MFS syndrome. A targeted-oriented therapy should include psychological evaluation of patients for anxiety and depression and pain management and fatigue, as research shows that these are important factors in the quality of life of patients, to alleviate negative psychological and physiological effects of stress in the early posttreatment period.

This study shows some shortcomings. First, since this was a single-center, retrospective study, there may have been selection bias. Therefore, we plan to verify the conclusions of this study by conducting a multicenter, large-sample, prospective randomized clinical trial. Second, because of the limited number of patients in our studies, we hope to cooperate with more hospitals in China to expand the sample size in the future.

## Conclusions

The QoL in young AAAD patients with MFS after surgery is lower than those without MFS. This may be associated with the uncontrollable persistent chronic pain and the uncertainty and concerns for the disease’s progression.

## Data Availability

The data that support the findings of this study are available on request from the corresponding author.

## Abbreviations

AAAD: acute type A aortic dissection
QoL: quality of life
SF-36: Medical Outcomes Study 36-item short-form health survey
HADS: hospital anxiety and depression scale
PCS: physical component summary
MCS: mental health component summary
